# Superwettable and injectable GelMA-MSC microspheres promote cartilage repair in temporomandibular joints

**DOI:** 10.3389/fbioe.2022.1026911

**Published:** 2022-09-20

**Authors:** Yue Yang, Chenyan Huang, Huimin Zheng, Zhaoqiang Meng, Boon Chin Heng, Tuanfeng Zhou, Shengjie Jiang, Yan Wei

**Affiliations:** ^1^ Beijing Laboratory of Biomedical Materials, Department of Geriatric Dentistry, Peking University School and Hospital of Stomatology, Beijing, China; ^2^ Department of Prosthodontics, The First Clinical Division, Peking University School and Hospital of Stomatology, Beijing, China

**Keywords:** superwettable, microspheres, temporomandibular disorder, chondrogenesis, spheroids

## Abstract

Temporomandibular disorders (TMD) can be treated by promoting cartilage regeneration with biomaterials. However, there are deficiencies in the infiltration function of bone filler biological materials. In this study, stems cells were loaded onto gelatin methacryloyl (GelMA) hydrogel microspheres endowed with superwettable properties and TGF-β sustained-release function, which can quickly infiltrate the irregular surface of the temporomandibular joint (TMJ) bone defect area and accelerate cartilage healing. First, to improve cell adhesion and spreading function, the BMSCs-coated GelMA microspheres were endowed with superwetting property. At the same time, the swelling adsorption characteristics of gelatin microspheres could be used to load recombinant TGF-β within the microspheres, which could in turn promote the chondrogenic differentiation of multi-potent bone marrow mesenchymal stem cells. The SEM imaging demonstrated that BMSCs-coated GelMA microsphere has superwettable and superhydrophilic property, which enabled rapid adaptation to the bone defect surface morphology, which is conducive to tissue repair. Furthermore, the cartilage defect model showed that rBMSCs-coated GelMA microspheres promote temporomandibular joint arthritis repair. In conclusion, our study established that BMSC-coated GelMA microspheres endowed with superwetting properties, can colonize the bone defect repair site better with sustained release of growth factors, thus providing an innovative strategy for promoting cartilage regeneration.

## 1 Introduction

Currently, tissue engineering approaches for treatment of temporomandibular joint disorders (TMD) are a much needed alternative to the limited efficacy of routinely-used clinical treatment modalities (Acri et al., 2019; Tarafder et al., 2016). Inducing differentiation of stem cells into chondrocytes within the condylar cartilage is a possible treatment strategy for TMD (Kim et al., 2023; Wadhwa and Kapila, 2008). However, biomaterials for cartilage regeneration within the temporomandibular joint (TMJ) is still in the developmental pipeline. The design of restorative materials with active infiltration capacity together with bio-inspired and biomimetic properties would be of great significance for the treatment of TMD.

The aim of biomimetic modification is to impart good biocompatibility to the base material, which facilitates good cytocompatibility and favorable osseointegration during the early stages of implantation (Del Bakhshayesh et al., 2019). However, current tissue repair materials often do not possess such properties, but often only provide early spatial occupancy and are usually not biologically permeable to the bone defect area. Most studies of supperwetting bioimplant materials focus on the infiltration characteristics of the base material (Jiang et al., 2022). As a natural extracellular-derived proteinaceous material, gelatin has been widely studied and applied in various biomedical fields, due to its implantability, biocompatibility and degradable properties (Stevens et al., 2002). However, gelatin itself as well as GelMA does not possess a biological infiltration capacity after implantation in the defect area, which limits its repair efficacy of bone defects (Lukin et al., 2022). Therefore, enhancing the bio-infiltration function of gelatin through surface modifications is expected to improve the biocompatibility and regeneration efficacy of implant materials.

Some studies have found that upon contact of mesenchymal stem cells (MSCs) with extracellular matrix (ECM) substrate, the infiltration and diffusion effect was similar to that of droplets touching the substrate (Douezan et al., 2011; Jiang et al., 2021). Combining the biological functions of MSCs with biomaterials to endow biomaterial with bio-inspired features is of great value for improving biocompatibility and optimizing the repair effect (Sulaiman et al., 2020). Stem cell microspheres have been extensively studied in recent years (Langhans, 2018). MSCs are a promising alternative for regenerative therapy because their lineage fate could be procisely regulated with specific growth factors (Han et al., 2019). Additionally, spheroid MSCs have shown higher therapeutic potential than MSCs monolayers through better spreading ability and increased secretion of growth factors (Cesarz and Tamama, 2016). On the other hand, there are also various difficulties in constructing cell spheroids, which often require the utilization of hydrogel scaffolds (Neto et al., 2016). Biomaterial microspheres can be used as individual cell culture units, or assembled into porous scaffolds or simulated biomimetic microenvironments, and have been widely applied in cell culture, tissue engineering, and regenerative medicine studies (Dhamecha et al., 2019). Hydrogels are good hydrophilic substrates, which are conducive to cell adhesion, and the hydrogel core can also promote the formation of MSCs spheroids (Cui et al., 2020; He et al., 2020). Surface wettability is an intrinsic but complex property of phospholipid bilayers, which can compensate for the deficiencies of artificial materials in adapting to the complex ultrastructural surface of irregular bone defects. Upon MSCs loading onto hydrogel microspheres, the cell membrane can serve as a natural substrate with strong affinity for endogenous stem cells, thereby optimizing the adaptability of the hydrogel to the TMJ defect surface ultrastructure. Being incorporated with the surface phospholipid bilayer and various cell membrane surface functional proteins, biomaterial based stem cell spheroids thus present superwetting and biological infiltration properties.

Additionally, the sustained-release of growth factors from hydrogels are also of great interest to tissue repair, in particular TGF-β (Bello et al., 2021). Previous studies have also found that sustained-release of growth factors (including TGF-β, FEGF, etc.) is an effective strategy to promote cartilage repair and treat TMD (Zheng et al., 2018). Moreover, one of the key reasons for the failure of 3D stem cell spheroid culture is the inability of growth factors to fully penetrate the spheroid core (Langhans, 2018). Using TGF-β-releasing microspheres as implantable scaffolds for the formation of cell spheroids is beneficial for maintaining the biological activity of spheroids, and directing chondrocyte differentiation, thus providing a promising strategy for tissue engineering-based treatment of TMD. Maintaining the sustained release of specific cytokines from the hydrogel internal core is a good strategy to guide the differentiation of stem cell spheroids.

**FIGURE 1:**
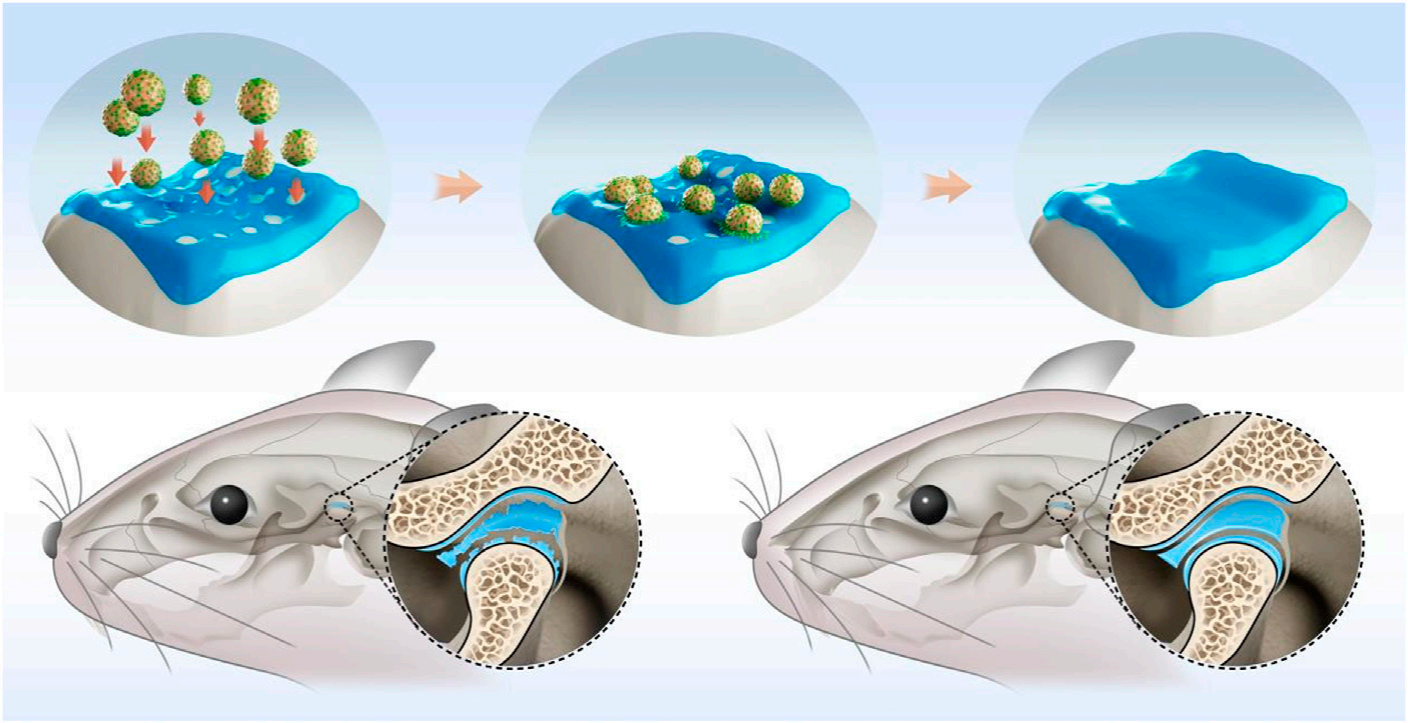
Schematic illustration of rBMSCs-coated GelMA microspheres promoting temporomandibular joint arthritis healing. GelMA: gelatin methacrylate; rBMSCs: rat bone marrow mesenchymal stem cells.

Here, we exploited the infiltration characteristics of MSCs by placing these cells on the surface of TGFβ-loaded GelMA microspheres, leading to active infiltration of biomaterial. The modified gelatin-MSCs microspheres can then more efficiently colonize the bone defect repair site, releasing cytokines at specific locations, and accelerating the healing of the TMJ defect area. Figure 1 depicts a schematic representation of the superwettable gelatin-MSCs microspheres for TMJ cartilage repair.

## 2 Materials and methods

### 2.1 Cell culture

Rat Bone Marrow-derived mesenchymal stem cells (rBMSCs) were cultured at 37°C under 5% CO_2_ atmosphere in αMEM enriched with 10% Fetal bovine serum (Procell). Upon reaching 80% confluency, cells were digested by 0.25% trypsin-EDTA (Procell) for 5 min. The cell suspension was centrifuged at 1,000 rpm for 10 min. After removing the supernatant, gently resuspend the cell and obtain a cell suspension.

### 2.2 *In vivo* animal experiment

All animal care and experiments were conducted under guidelines stipulated by the Animal Care and Use Committee of Peking University (LA2022175). 6-weeks-old male SD rats were randomly assigned into three groups: the BMSC-coated microspheres group, microspheres group and the control group. Rats were subjected to bilateral temporomandibular joint (TMJ) cavity injection of 50 µl complete adjuvant (Complete Freund’s Adjuvant) combined with 10 ng/ml IL-1β (Sigma) to establish the TMJ arthritis model. A week later, 200 μl BMSCs-coated microspheres, 200 μl blank microspheres or 200 μl saline were injected into the TMJ cavity. On day 7 and 14, rats were sacrificed and the defect lesions of condylar cartilage were extracted and fixed in 4% (w/v) paraformaldehyde for 24 h.

### 2.3 Fabrication of the rBMSCs coated microspheres

2 mg of GelMA microspheres (EFL, Suzhou, China) were added to each well of a 24-well culture plates, and the culture plate was sterilized by ultraviolet light in a biological safety cabinet. 30 min later, 500 μl of culture medium was added to each well of the culture plate. The microspheres in a 37°C incubator for 30 mins for swelling. The morphology of swelling GelMA microspheres were confirmed. (Supplementary Figure S1, Supporting Information). rBMSCs suspension was added to each well and mixed with the microspheres. Observations under optical microscopy showed that rBMSCs were uniformly adherent to the surface of GelMA microspheres after 24 h incubation (Supplementary Figure S2, Supporting Information).

### 2.4 Scanning electron microscope

#### 2.4.1 Microspheres sample preparation

As described above, rBMSCs coated microspheres were obtained and then wash three times with 4°C prechilled PBS for 5 min each. After aspirating PBS, add 2.5% isopropanol at 4°C for 2 h. Wash three times at 4°C pre-chilled PBS for 5 min each. The microspheres were dehydrated with alcohol gradient (20 mins each in 50, 60, 70, 80, 90% and 30 mins in 100%). Finally, critical point drying was performed. The Hitachi MC1000 Ion Sputter Coater was used to spray gold on the surface of the material for 120 s. Scanning electron microscopy (SEM) images were captured in the field-emission mode using a FEI Quanta 450FEG (USA) at an acceleration voltage of 5 kV.

#### 2.4.2 Animal samples

Extracted temporomandibular joints were dehydrated in an alcohol gradient followed by drying in vacuum. Images were captured in the field-emission mode using a FEI Quanta 450FEG (USA) at an acceleration voltage of 10 kV.

### 2.5 Immunocytochemistry

The microspheres coated with BMSCs were seeded in matrigel (Corning) for 12 h, fixed in a 10% (w/v) formalin solution for 10 min, washed with PBS, and permeabilized with 0.1% (w/v) Triton X-100 in PBS for 10 min. Then, the cells were incubated with rabbit anti-SOX9 (ab185966, Abcam) and rhodamine-phalloidin in PBS with 0.3% (w/v) Triton X-100 and 3% (v/v) donkey serum for 2 h. Cell nuclei were stained with 1 μM DAPI for 10 min.

### 2.6 ELISA

#### 2.6.1 Sample collection

GelMA microspheres were incubated in αMEM or chondrogenesis induction medium for 24 h and then the supernatant was aspirated. 500 μl of PBS was added to each well, and the supernatant was collected for 1, 3, and 6 h, respectively.

#### 2.6.2 ELISA assays

The rat TGF-β ELISA kit (MEIMIAN, Jiangsu, China) was used to assay the TGF-β levels in the sample. 50 μl aliquots of standards with different concentrations of TGF-β were added to each well of a 96-well plate; Then 10 μl of the sample to be tested were added to each well, together with 40 μl of buffer for sample dilution. And the reaction wells were sealed with sealing plates and incubated at 37°C for 30 min. After incubation, the supernatant liquid was then discarded and the sample wells were patted dry on an absorbent paper. Washing with washing solution was repeated five times. In addition to the blank wells, 100 μl of HRP-conjugated reagent was added to each well. Washing was repeated five times. Then, 50 μl of chromogen solution A and 50 μl of chromogen solution B were added into each well and incubated at 37°C for 10 min, in the dark. Then 50 μl of the stop solution was added to each well, and the OD value of each sample was measured at 450 nm with microplate reader (LP400).

### 2.7 Real-time quantitative reverse transcription PCR

Total RNA was extracted using TRIzol (Thermo Fisher), chloroform and isopropyl alcohol. Reverse transcription was performed using a PCR thermal cycler (Takara). Optical 96-well reaction plates (Thermo Fisher Scientific) and optical adhesive films (Thermo Fisher Scientific) were used for the PCR. The PCR mixture loaded in each well had a final volume of 10 µl, and included 5 µl FastStart Universal SYBR Green Master Mix (Rox), 3 µl RNase-free water, 1 µl template cDNA, and 1 µl primer. PCR amplification was conducted with the following cycling parameters: 15 min at 95°C (heat activation step), followed by 40 cycles of 15 s at 95°C and 1 h at 60°C. Data were analyzed using the standard curve method and normalized to GAPDH mRNA levels.

**TABLE 1:** Primer sequences utilized for qRT-PCR.

Gapdh	TCT​CTG​CTC​CTC​CCT​GTT​C	ACA​CCG​ACC​TTC​ACC​ATC​T
Sox9	GAA​AGA​CCA​CCC​CGA​TTA​C	TGA​AGA​TGG​CGT​TAG​GAG​A
Col2α1	GACGCCACGCTCAAGTC	TCT​CCG​CTC​TTC​CAC​TCT​G
Aggrecan	CCC​AAA​CAG​CAG​AAA​CAG​C	GGT​GGC​TCC​ATT​CAG​ACA​A

### 2.8 Immunohistochemical staining

The dissected temporomandibular joints were embedded in paraffin after decalcification. Serial sections subjected to immunohistochemistry staining by using rabbit anti-rat Sox9 mAb, hematoxylin and eosin. For SOX9 staining, a biotinylated anti-rabbit IgG secondary antibody and streptavidin-Horseradish peroxidase (HRP), followed by colorimetric detection using DAB.

### 2.9 Microcomputed tomography analysis

After the macro evaluation, the specimens were examined using a Viva40 micro-CT (Scanco Medical AG^®^). After scanning, the appropriate sagittal and coronal cross-sections of temporomandibular joints were adjusted in the software, and 3D reconstruction was performed on each femur sample. Then a columnar region of interest (ROI) (diameter 3.5 mm, height 1 mm) was selected at the sample defect regeneration area. All analyses were performed on the digitally extracted callus tissue using 3D distance techniques (Scanco^®^ software).

### 2.10 Statistical analysis

Numerical data were presented as mean ± SD. To evaluate the significance of observed differences between the experimental groups, the one-way analysis of variance (ANOVA) and Student t-test were used. A value of *p* < 0.05 was considered to be statistically significant.

## 3 Results and Discussion

### 3.1 Fabrication of superwettable BMSC-coated microspheres

**FIGURE 2:**
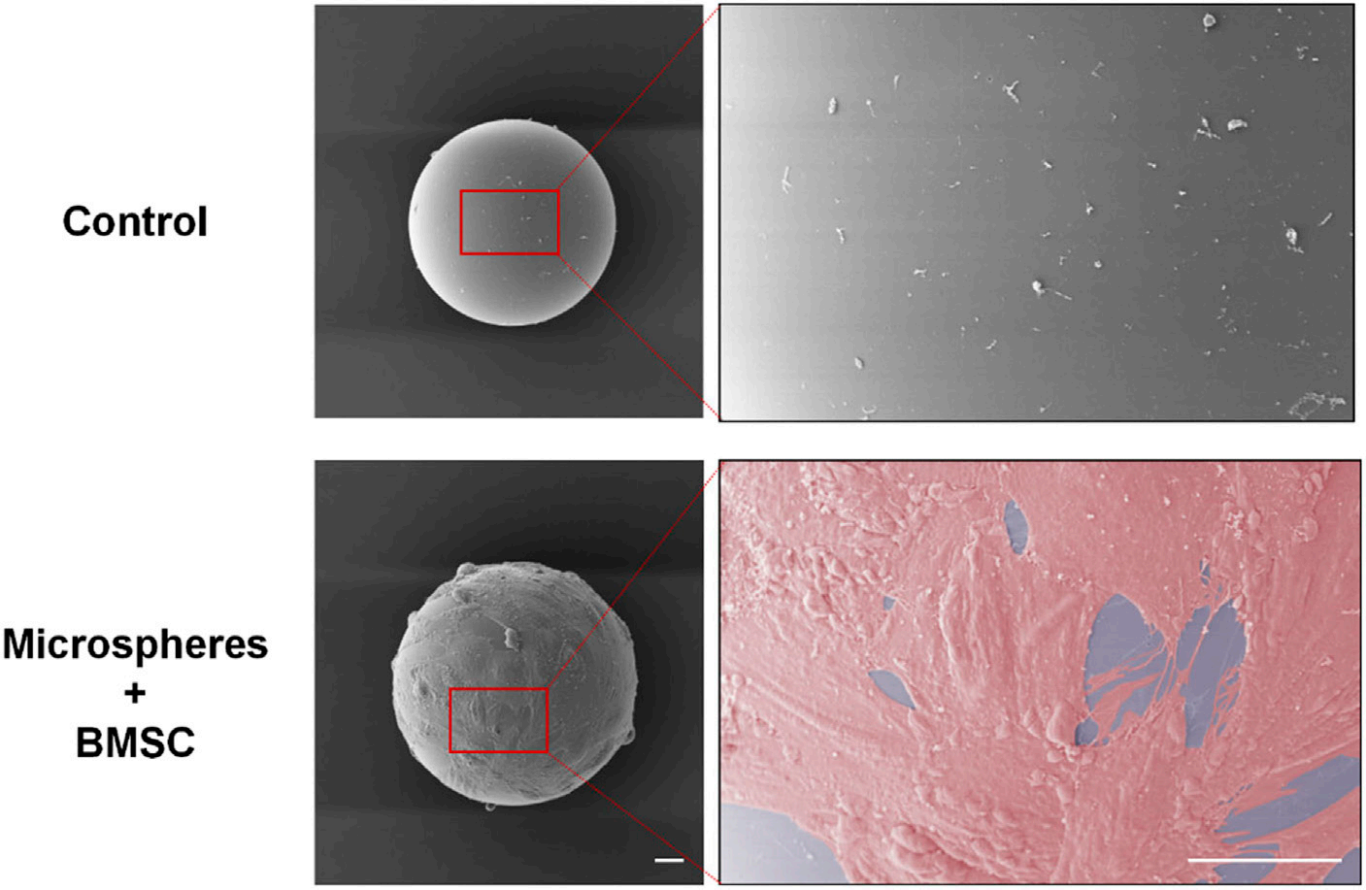
The rBMSCs cultured with swelled and disinfected microspheres for 24 h. SEM imaging results showed that BMSCs adhered to the surface of the microspheres and spread well on the hydrogel surface. Scale bars = 20 μm. rBMSCs, Rat bone marrow mesenchymal stem cells.

First, we constructed the GelMA microspheres coated with rBMSCs. GelMA microspheres swelling and the adherent of rBMSCs to the microspheres surface were confirmed (Supplementary Figure S1–3, supporting information). The SEM imaging results showed that BMSCs could tightly adhere to the surface of the microspheres and spread well on the hydrogel surface (Figure 2). The cell spreading also suggested that the GelMA microspheres have good biocompatibility, which could contribute to the formation of BMSC spheroids. The complete coating of microspheres with BMSCs provided a stable phospholipid bilayer at the outermost layer of the microsphere, and its hydrophilic properties are beneficial for adapting to the ultrastructure of the defect surface, thereby enhancing the repair of complex cartilage dysfunction caused by friction, inflammation, and other reasons. It was found that upon contacting the ECM substrate, the diffusion and spreading of stem cell loaded microspheres were similar to that of droplets touching the substrate (Chatzinikolaidou, 2016; Dhamecha et al., 2019). Compared to conventional cell culture methodology, the microspheres have a higher specific surface area, and the multi-microsphere assembly scaffold has an interconnected porous structure, which provides more space for cells to proliferate and differentiate, thereby accurately simulating the natural tissue microenvironment (He et al., 2020; Wang et al., 2023). In our study, by fabricating the BMSC-coated hydrogel microsphere, the biomaterial incorporated with the cell phospholipid bilayer attains superwetting property that facilitates tissue spreading at the ultrastructural level. Furthermore, based on their regulatable and injectable properties, they can be utilized for minimally-invasive treatment of irregular wounds at the ultrastructural level.

### 3.2 Characterization of the superwettable GelMA microspheres

**FIGURE 3:**
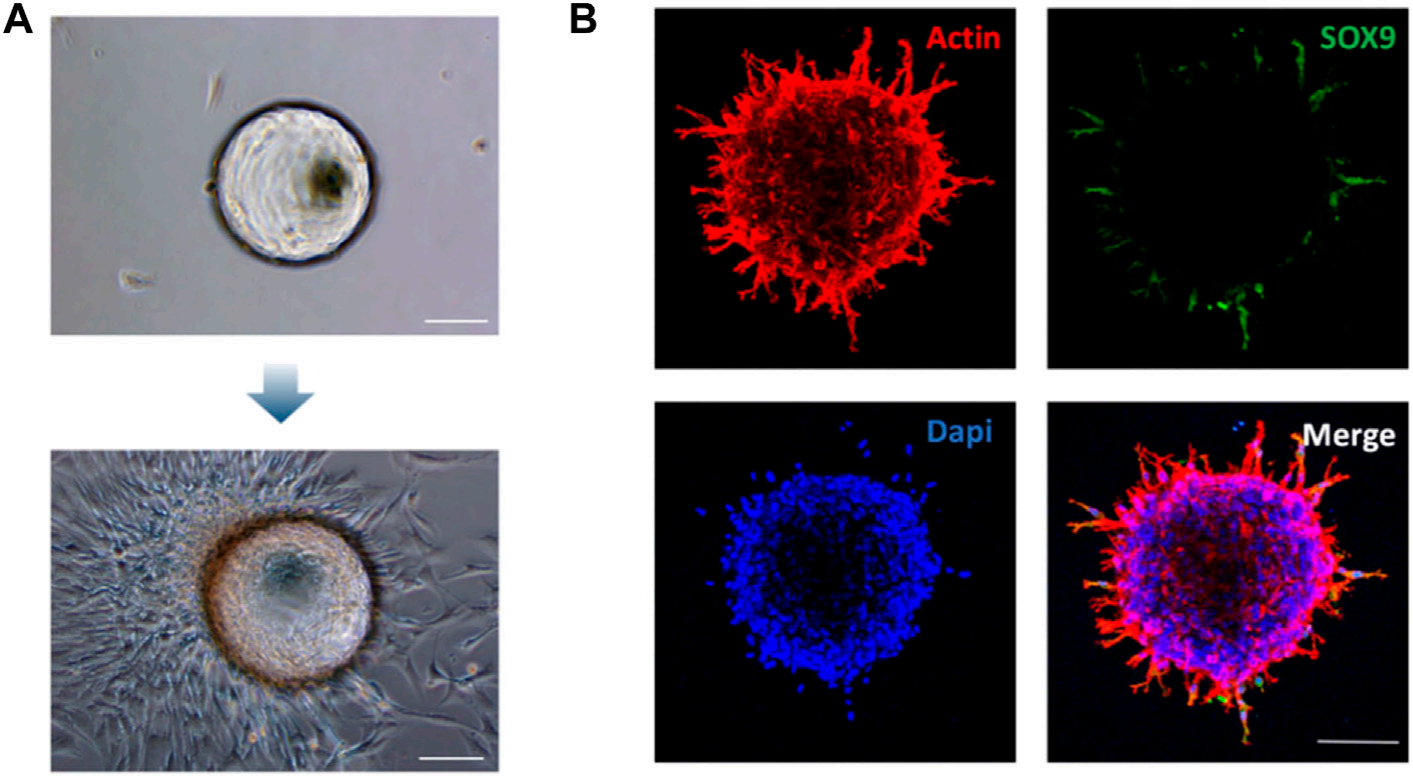
Morphological and phenotypic characterization of the rBMSCs-coated GelMA microspheres. **(A)** Identification of rBMSCs spheroid morphology coated on Gelatin Microspheres on day 7. **(B)** Immunocytochemical staining showing the expression of chondrogenic marker Sox9 on the rBMSCs spheroid at 72 h. Scale bars = 100 μm.

The molecular and biological characteristics of BMSCs-coated microspheres were analyzed to verify the chondrogenic differentiation potential. GelMA microspheres coated with rat BMSCs were transferred to six-well plates for culture. Observations under optical microscopy showed that after 7 days of culture, the BMSCs-coated microspheres tended to spread on the bottom of the culture plates, which resulted in tight adhesion of the microspheres to the bottom of the culture plates (Figure 3A). It is well-known that SOX9 transcription factor is required for chondrocyte differentiation and cartilage formation (Behringer et al., 1999; Lefebvre and Dvir-Ginzberg, 2016). The immunocytochemistry results showed that the chondrogenic marker SOX9 was highly expressed (Figure 3B). These results thus indicated that the rBMSC-coated GelMA microspheres presented good adhesion potential, which could provide a biological basis for the subsequent repair of the tissue defect area. The expression of Sox9 suggested that the rBMSCs-coated GelMA microspheres exhibit chondrogenic potential for cartilage defect repair. The above results thus showed that promotion of the formation of superwettable spheroids by microspheres is an effective and innovative bioengineering strategy to facilitate TMJ cartilage regeneration and treatment of related disorders.

### 3.3 Sustained release of TGF-β and chondrogenic potential of BMSC-coated gelatin methacryloyl microspheres

**FIGURE 4:**
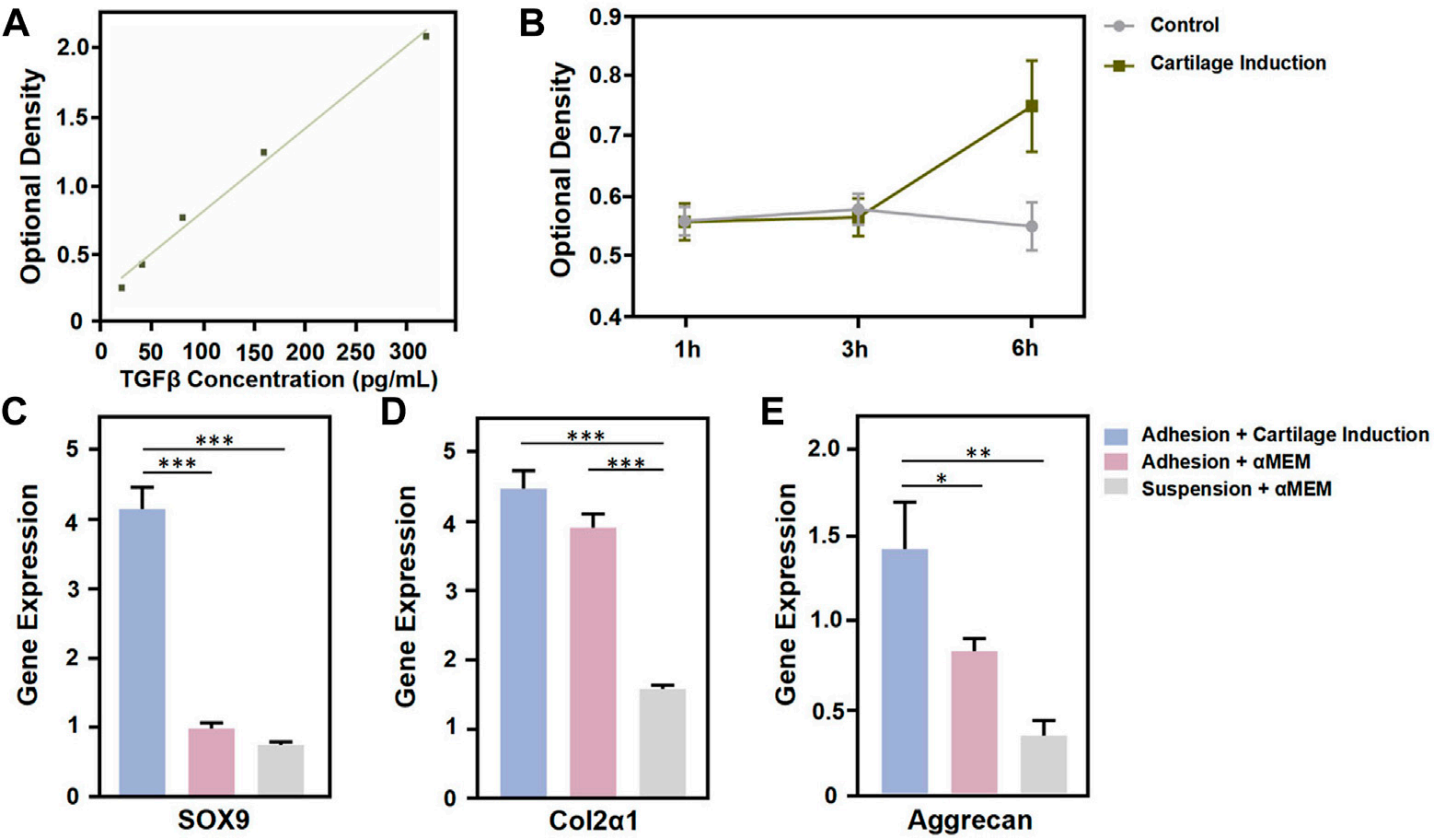
Functional characterization of GelMA Microspheres. **(A)** TGF-β concentration was determined using an ELISA kit. **(B)** Detection of TGF-β release in the control and chondrogenic induction medium group at 1 h, 3 h and 6 h. **(C, D, E)** Total RNA from cells coated to microspheres of each group was subjected to quantitative real-time PCR after 72 h culture. Relative expression of the indicated genes against GAPDH is shown. *n* = 4 for each group. (*p < 0.05, **p < 0.01, ***p < 0.001). All the results were repeated three times and represents means ± SD.

There are many technical difficulties faced in the cultivation of cell spheroids (Baraniak and McDevitt, 2012; Chatzinikolaidou, 2016). Microspheres can be assembled into porous scaffolds or simulated biomimetic microenvironments, and have been widely used in cell spheroid formation for tissue engineering and regenerative medicine applications (He et al., 2020). Furthermore, microspheres can be used extensively as delivery vehicles for cells and drugs, particularly in providing more conducive proliferation and differentiation conditions for cellular spheroids, by accurately simulating natural tissue microenvironments. It has been reported that the cell growth factor TGF-β can act synergistically with various cell types to promote the healing of defect sites (Bello et al., 2021). The swelling capacity of GelMA microspheres facilitates the loading and continuous sustained release of cytokines, thereby enabling the cell spheroids to regulate differentiation and tissue repair (Nii, 2021). In this study, similar to the use of PBS, microsphere swelling can also be achieved by soaking GelMA microspheres in culture medium containing recombinant TGFβ. Then the TGF-β release profile was characterized by placing the swelled microspheres into blank PBS. The ELISA results showed that the TGF-β concentration was linearly positively correlated with the OD value (Figure 4A). The OD values corresponding to TGF-β concentrations in the two groups were basically similar during the first 3 h, as compared to the aMEM group, while the concentration of TGF-β released from microspheres in the chondrogenic induction medium group increased significantly by the 6 h timepoint. This results demonstrated that prior to loading with BMSCs, the gelatin-microspheres can be endowed with TGF-β releasing capacity by incubating them with culture medium containing TGF-β. The sustained release of TGF-β from GelMA microspheres would be more conducive to the repair of tissue defects. In order to investigate the sustained TGF-β release effects on the promotion of chondrogenesis, the cell phenotype was examined. The microspheres were assigned to three groups: chondrogenesis-induced adhesion group, αMEM adhesion group and αMEM suspension group. The results showed that the chondrogenesis-related gene markers SOX9, Col2α1, and Aggrecan were all highly expressed in the chondrogenesis-induced adhesion group after 72 h culture (Figures 4C–E). These results thus demonstrated that the BMSCs-gelatin microspheres enabled rapid cell spreading, as well as the loading and sustained release of bioactive factors that can contribute to cartilage healing.

### 3.4 rBMSC-coated gelatin methacryloyl microspheres promote cartilage repair in a rat temporomandibular defect model

**FIGURE 5:**
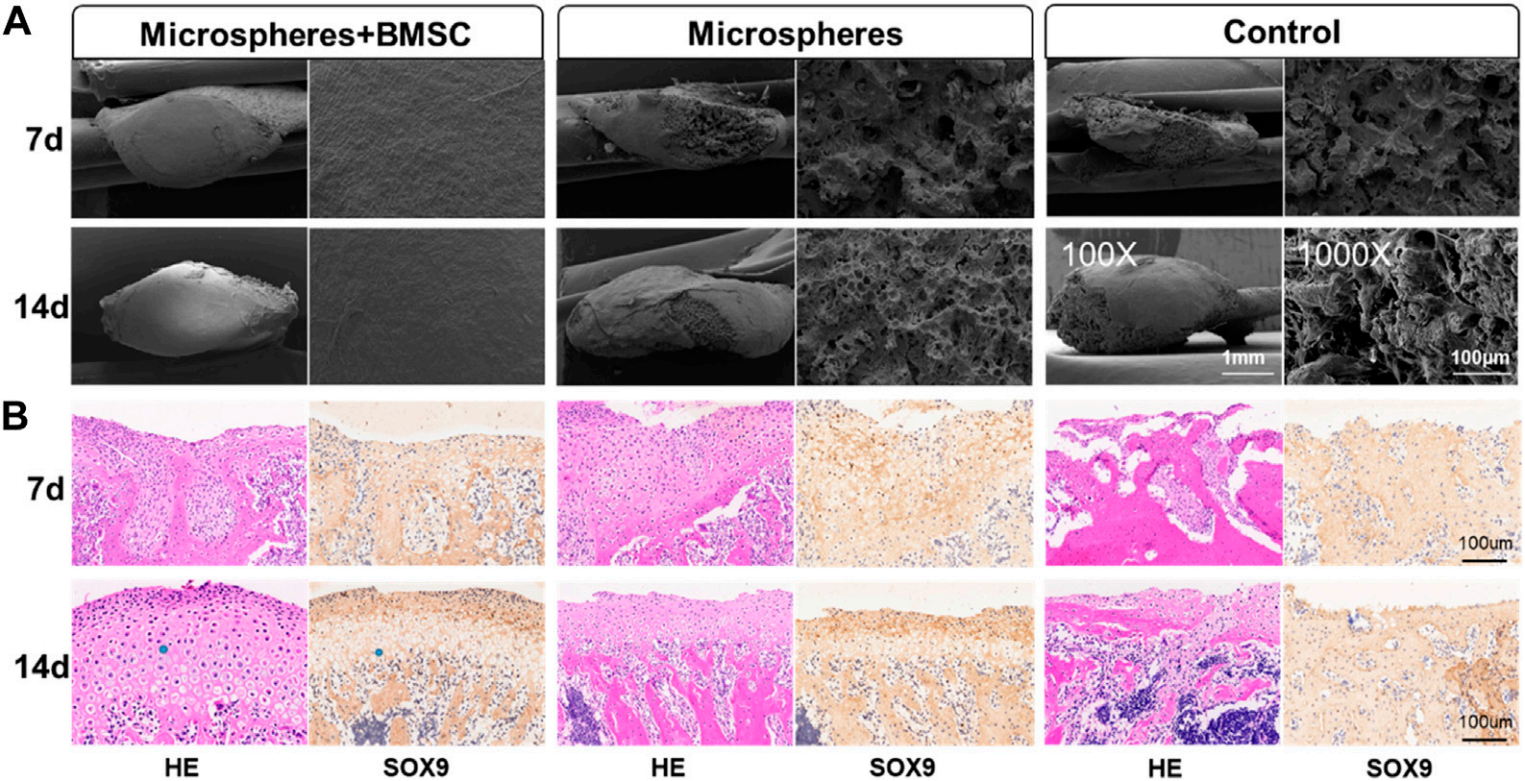
rBMSCs-coated GelMA microspheres promote temporomandibular joint arthritis repair. **(A)** SEM of condyle articular surface of TMJ. The microstructure of TMJs were detected by a scanning electron microscope (SEM) under the magnifications: ×100, and ×1,000. **(B)** HE and immunohistochemical staining of TMJ defect area. Data are representative from each group of six rats and all the results were repeated three times. SEM, scanning electron microscope; TMJ, temporomandibular joint.

**FIGURE 6:**
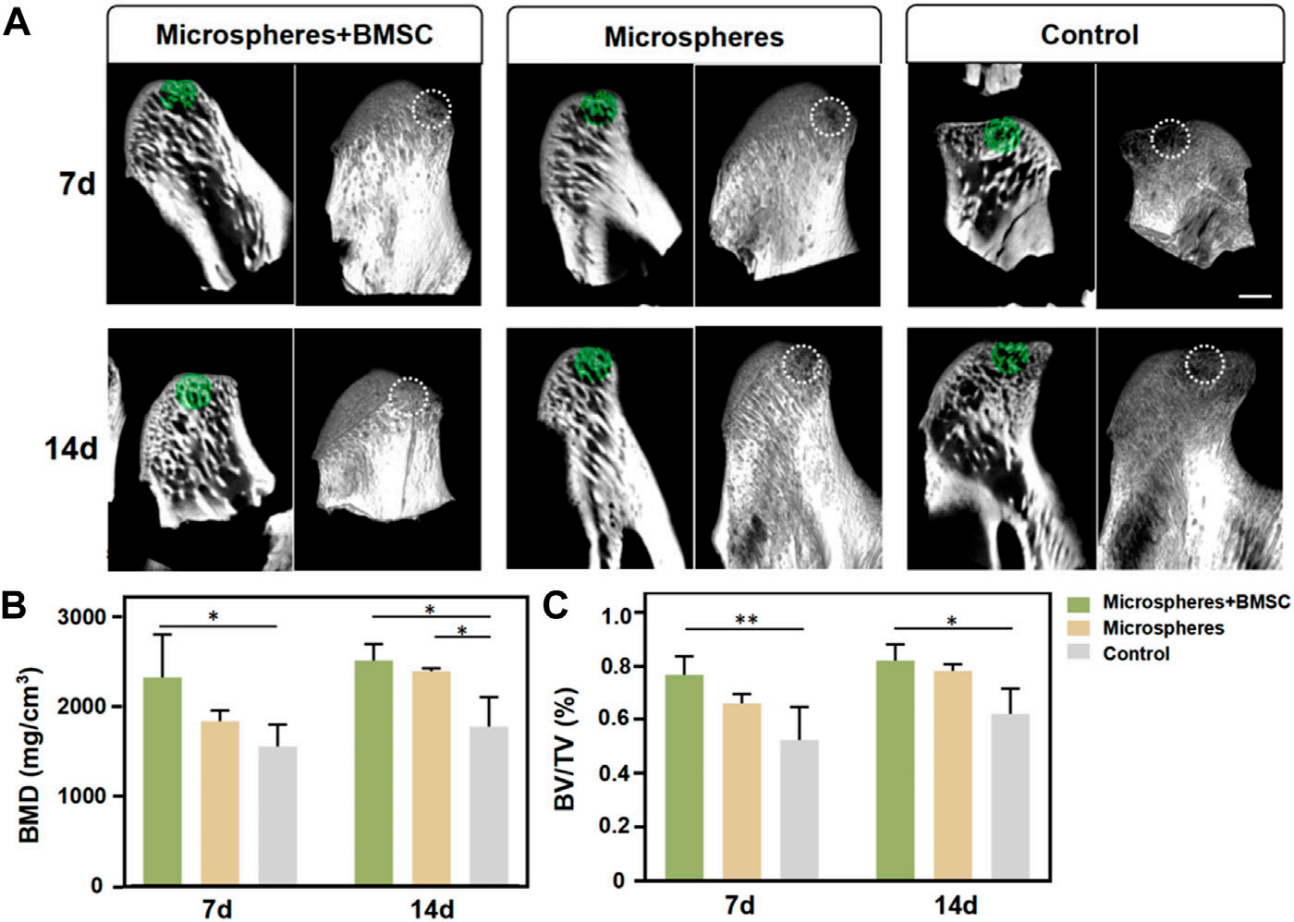
Micro-computed tomography (micro-CT) evaluation. **(A)** Micro-CT of temporomandibular joint defect area. **(B)** BMD and **(C)** BV/TV within the ROI were determined. Scale bar = 1 mm. Data are representative from each group of six rats. (*p < 0.05, **p < 0.01, ***p < 0.001).

Utilizing a rat TMJ cartilage defect model, we further verified the function of BMSCs-coated GelMA microspheres in promoting cartilage repair *in vivo*. As shown in Figure 5A, the SEM imaging results indicated that the temporomandibular joint defect in the rBMSCs-coated GelMA microsphere group was almost completely healed, with the healed surface being smoother than the other two groups. HE and immunohistochemical staining results of the tissue sections showed that the formation of new bone and cartilage within the temporomandibular joint of the rBMSCs-coated GelMA microsphere group was significantly better than that of the other two groups (Figure 5B). The results of the Micro-CT also indicated that the rBMSCs-coated GelMA microsphere group had more complete bone cortex and denser trabecular bone within the temporomandibular joint (Figures 6A,B). Overall, chondrogenesis-induced rBMSCs-coated GelMA microspheres displayed positive efficacy in promoting cartilage repair in temporomandibular arthritis. Future research will further explore the underlying mechanisms to lay a solid foundation for clinical applications.

## 4 Conclusion

In summary, we established cell spheroids with superwetting properties *via* coating of GelMA microspheres with BMSCs. The formation of cell spheroids was better facilitated by using a biocompatible hydrogel as the core. After the cells have spread and were tightly adherent to the surface of the hydrogel sphere, the phospholipid bilayer of the cell membrane conferred on the cell-coated microsphere better hydrophilicity and tissue adhesion properties, thereby facilitating the repair of irregular cartilage defects *in vivo*. At the same time, the inner GelMA microspheres can also be utilized for continuous and sustained release of recombinant TGF-β, which enhanced spheroid differentiation and chondrogenesis. The *in vitro* results showed that the cell spheroids strongly expressed gene markers related to cartilage differentiation. Additionally, the *in vivo* TMJ cartilage defect repair experiments also demonstrated that BMSCs-coated microspheres can effectively promote the repair and reconstruction of irregular cartilage defects within the TMJ area. This study thus provides a novel strategy for fabricating biomaterial-cell construct systems with superwetting microstructures that is beneficial for tissue repair in TMD and other related diseases.

## Data Availability

The original contributions presented in the study are included in the article/Supplementary Material, further inquiries can be directed to the corresponding authors.
