# Supplementary material for: Superwettable and injectable GelMA-MSC microspheres promote cartilage repair in temporomandibular joints
Source: Front Bioeng Biotechnol. 2022 Sep 20;10:1026911. doi: 10.3389/fbioe.2022.1026911 (PMC9549523; doi:10.3389/fbioe.2022.1026911)
Supplement: Supplementary file 1 [file DataSheet1.docx]

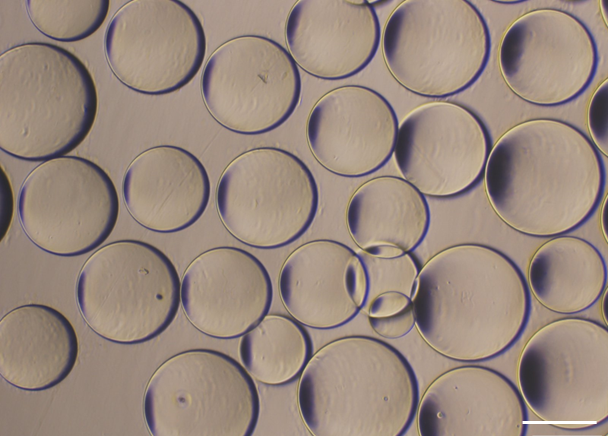


**Supplementary figure1** Optical microscopy imaging showed that the diameter of each microsphere ranged from 150μm~300μm.

Scale bar = 200μm


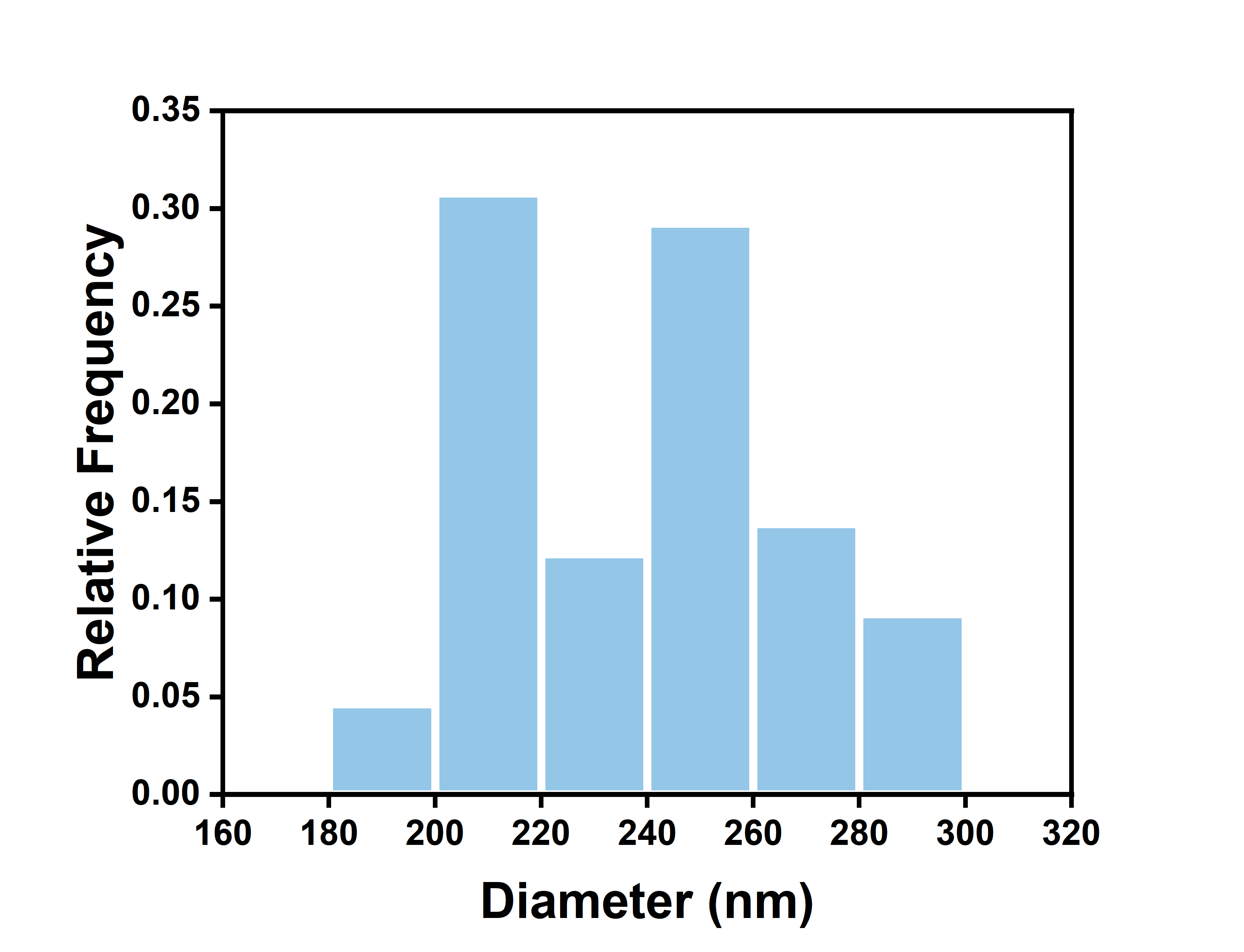


**Supplementary figure2** The total number of 100 swelling GelMA microspheres were randomly selected and the diameters were measured.


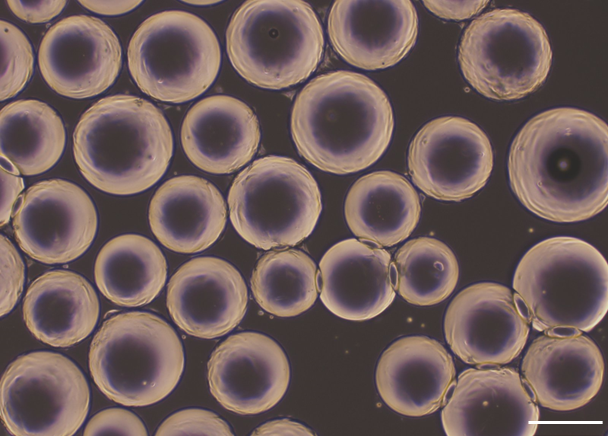


**Supplementary figure3** Observations under optical microscopy showed that rBMSCs were uniformly adherent to the surface of GelMA microspheres after 24h incubation.

Scale bar = 200μm
